# Utilizing Instagram Stories to deliver educational content to medical students in pediatrics: curricular design and evaluation

**DOI:** 10.1080/10872981.2025.2534064

**Published:** 2025-07-18

**Authors:** Raymond Bellis, Rachel Kim, Rebecca Lendway, Courtney Sniffen, Nathan Smith, Maribeth Chitkara

**Affiliations:** a Stony Brook Medicine, Department of Ophthalmology, Stony Brook, NY, USA; b Department of Pediatrics, University of California Department of Pediatrics, San Francisco, San Francisco, CA, USA; c Stony Brook Children’s, Renaissance School of Medicine at Stony Brook University, Stony Brook, NY, USA; d Comer Children’s Hospital, University of Chicago, Chicago, IL, USA

**Keywords:** Undergraduate medical education, Instagram, social media, pediatric clerkship, Technology in education

## Abstract

Utilization of social media has been described in medical education, but Instagram as an educational platform for the pediatric clerkship has not yet been reported. We created a 29-item Instagram Story curriculum with summative examination-relevant topics to supplement the pediatric clerkship for third-year medical students. Engagement in the Instagram curriculum was assessed by measuring Storyviews, calculating Story retention rates (number of users who viewed al lslides/number of users who viewed the first slide  100) and analyzing mini-quizperformance.  Students completed a voluntary 7-item survey at clerkship end to assess overall satisfaction with the Instagram curriculum.  Quantitative analysis of questions using a 7-item scale and qualitative analysis of responses from open-ended questions were performed. A total of 132 students participated over a period of eight 6-week blocks.  During the study period, the average number of views on the first slide in a Story was 24.8 and the average number of views on the last slide of a story was 13.3 per block.  Average retention rate was 54.3%, mini-quiz engagement rate was 32.0%, and percent correct on mini-quizzes was 87.6%.  Students positively rated the Instagram curriculum on feasibility (83.6%), clerkship relevance (89.1%), facilitating engagement in the material (65.5%), assisting in NBME pediatric subject exam preparation (61.8%), and recommending the modality to other clerkships (69.1%). This study provides evidence that the use of an Instagram curriculum within a core pediatric clerkship is feasible, engaging, relevant, and favorably received by medical students.  We conclude that Instagram can be successfully integrated into clinical undergraduate medical education.

## Background

Healthcare education is in the midst of a profound transformation, fueled by unprecedented knowledge and informational growth. Curricular reforms in undergraduate medical education (UME) have seen more integration of basic science with clinical learning, flipped classroom teaching, and self-directed learning [1]. Medical educators struggle to deliver content to students in a meaningful way with attendance at traditional lectures waning [2,3]. Studies of pre-clinical medical students have demonstrated a preference for self-directed learning over the classroom, even opting for viewing of recorded lectures over in-person attendance [4]. A 2020 study of medical students from a variety of training programs in the U.S. sought to gain a deeper understanding of their learning needs and motivations by engaging in a series of small group interviews [5]. One area of reform identified was specific to the use of technology in medical education, with students expressing interest in being able to source their own learning materials to supplement those provided by a professor. The investigators noted that students were extremely facile with novel technology and subsequently availing themselves of alternative sources of information, expressing frustration with the more traditional educational didactic model [5].

With the introduction of Web 2.0, internet-based social media applications emerged that allowed for the creation and exchange of content among users, the most popular including Facebook, YouTube, Instagram, X (formerly known as Twitter), and TikTok. While usage of social media cannot be considered ubiquitous, it could certainly be described as widespread. More than 60% of the world population engages in various forms of social media, with average daily time spent surpassing two hours [6]. According to 2025 estimates, there are currently 1.74 billion Instagram users, with the highest population of these users between the ages of 16–34, correlating to years spent in college and medical school [6,7]. Al Faris et al. reported that 90% of college students access social media, 72% of students have a personal social media profile and 45% use that profile at least once per day [7]. These platforms encourage users to communicate and engage with one another through the sharing of information, comments, and messages, often in real-time. As a result, they have become widely used by students as an alternative approach to supplement traditional learning strategies [7].

Amid the COVID-19 pandemic, the use of social media in medical education expanded rapidly as educators began to embrace the need to deliver curricular materials in a remote, asynchronous method. Social media platforms afforded teachers and learners the ability to engage with each other in learning communities and establish camaraderie that might otherwise not have been possible due to social distancing limitations [8]. A Canadian institution developed a resident-led Instagram account that included multiple medical specialties with graduate medical trainees posting teaching cases on a weekly basis to supplement student education during remote learning in the height of the COVID-19 pandemic. Investigators noted a five-fold increase in the reach of posts over the course of the academic year following account creation, speaking to its popularity among learners [8]. This type of initiative allows for both growth of the graduate trainee as an educator and facilitation of mentor-mentee relationships among learners of different training levels.

Social media use in medical education has been reported in a variety of disciplines, including radiology, obstetrics and gynecology, hematology-oncology, orthopedic surgery, and graduate medical education in general [8–17]. A 2019 systematic review of 53 publications regarding social media use among residents identified 14 with content specific to medical education. Up to 97% of trainees in the included studies reported using social media for personal purposes and 77% used social media platforms to bolster their medical education [14]. Use of social media in UME specifically has been described in preclinical courses such as anatomy, cardiology, hematology/oncology as well as advanced educational endeavors such as residency preparation and professionalism training [18]. Despite its popularity among UME students, there have been no studies published regarding the use of social media for the delivery of educational content in the core pediatric clerkship to date.

Core clinical clerkships are charged with providing quality clinical and didactic educational experiences as well as adequate preparation for a summative exam at the completion of the rotation. Many clinical clerkships utilize the standardized National Board of Medical Examiners (NBME) pediatric subject exams, also known as shelf exams, as summative objective clerkship assessments [19]. The shelf exam provides a means of equalizing expectations of knowledge acquisition across the span of an academic year within and among institutions, which is particularly important in pediatrics given the seasonal nature of cases encountered. This summative activity requires students to spend a significant amount of time outside of the clinical environment searching for and engaging in NBME-relevant, high-yield, and reliable preparatory materials. With this study, we sought to introduce a supplementary educational program to students on the pediatric clerkship utilizing social media, designed to address both their clinical and summative examination learning needs.

Instagram is an ideal platform to share teaching points and engage learners in an asynchronous manner by virtue of its use of visually appealing graphics. Instagram posts can be static; saved to the feed of the account holder, or active using the ‘Instagram Stories’ modality. Static posts remain present in the user’s social media feed, enabling them to scroll through the content at their leisure to consider the teaching material. The active functionality of Instagram Stories complements the more traditional static social media posts and are designed to be fast, memorable, and fun. Stories allow for a series of images or videos to appear on an account’s feed that is only visible to the user for a 24-hour period. Each slide in a Story can be used to deliver content using both graphics and text with a time limit of 60 seconds per Story. The Story feature allows the creator to embed short quizzes and polls into the content, enabling users to directly engage with the Story. The content can be curated in a way that provides the user with immediate and direct feedback. Engagement with the Story can be measured and tracked over time. Definitions of social media engagement in the literature vary and depend on the mode of social media used, ranging from measurements of account followers, views, likes, or comments in response to posts [12].

The primary objectives of this study were to: 1. build a library of pediatric NBME-relevant content for students on their core clerkship in pediatrics, 2. utilize the Instagram platform for dissemination, using the Story functionality in particular, and 3. evaluate engagement and overall satisfaction with the Instagram curriculum by medical students rotating on the core clinical clerkship in pediatrics.

## Methods

### Instagram Curriculum Development

**Figure 1.**
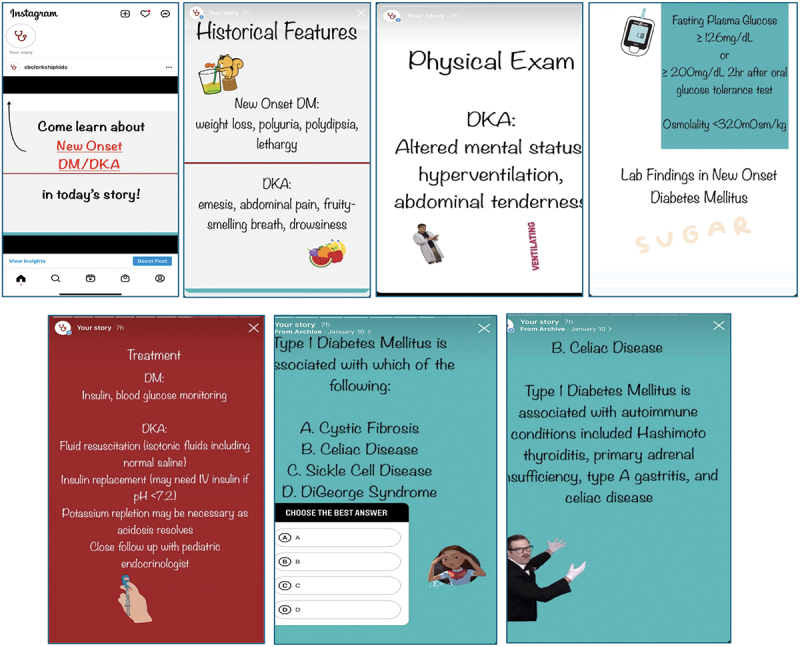
Sample pediatric Instagram Story.

The SBClerkshipKids (Knowledge in Doses) Instagram account was developed by the authors (RB, RK, RL, CS, NS, and MC) to deliver the curricular content. Twenty-nine different Instagram Stories were created, covering a range of core pediatric topics such as anemia, asthma, and neonatal rashes. Topics were selected based on investigator consensus and included input from three senior medical students and two pediatric senior residents, each with recent experience as both clerkship students and NBME examinees. Amenability of content delivery via the Instagram Stories platform was also considered in topic selection, thereby identifying subjects best taught with graphics such as images of acute otitis media or radiographs of intussusception. Study investigators grouped Stories of related topics into weekly themes (for example, one week’s Stories had a respiratory focus featuring croup/bronchiolitis/asthma/epiglottis/foreign body aspiration). The same schedule of Story topics was followed for each new clerkship cohort of students (Table 1). Stories consisted of up to 10 slides containing information on each topic organized by historical features, physical exam, diagnostic studies, and treatment. Each Story ended with an interactive multiple-choice question that users could answer prior to viewing the correct answer choice and explanation, followed by a slide with references for the information and images found in the Story (Figure 1). Images were secured from Wikimedia Commons, an online repository of 100 million freely usable images. Due to time limits intrinsic to Instagram, each Story was limited to 60 seconds to review. Learners were able to slow the pace of the Story by tapping and holding an image to regard it for a longer period of time.

**Table 1.** Example schedule of pediatric Instagram Story 6-week block curriculum.

Schedule of Story Topics					
Week	Monday	Tuesday	Wednesday	Thursday	Friday
1	Meckel Diverticulum	Otitis Media	Milk Protein Allergy	Anemia	Renal abnormalities/ posterior urethral valves
2	Viral Acute Gastroenteritis	Pyloric Stenosis	Bilious Emesis	Intussusception	Celiac Disease
3	Croup	Bronchiolitis	Asthma	Epiglottitis	Foreign Body aspiration
4	Juvenile Idiopathic Arthritis	Henoch Schonlein Purpura	Kawasaki Disease	Systemic Lupus Erythematosus	Hemolytic Uremic Syndrome
5	Eczema	Acne	Newborn rashes	TORCH infections	Rashes: Hand Foot Mouth, Roseola, measles, parvovirus
6	Trisomy 21/13/18	New onset Diabetes/ Diabetic Ketoacidosis	Cystic Fibrosis	Sickle Cell Anemia	OFF (Shelf Exam)

All curricular content on the account was reviewed and approved by a Stony Brook Children’s senior faculty member (MC). Each weekday, a new Story was posted (Table 1), and each weekend, summary slides were posted on the Instagram account reviewing topics from the prior week. These summary posts were static in nature, and as such could be reviewed by students after the Story content had disappeared from the account. On occasion, the formatting of Stories was altered accordingly if issues were noted regarding accessibility/readability (e.g., font size, color, or quantity of information per slide) based upon real-time user feedback.

### Study Design and Curricular Analysis

The study period occurred across eight 6-week clerkship blocks. The participants involved in the study were medical students recruited from the pediatric clerkship cohorts and spanned two separate academic years. Data collected from the Instagram account included the number of views for each slide and the number of responses (both correct and incorrect) to each question posted. As the Instagram account was public, there were often users outside the target participants (e.g., authors, Stony Brook Children’s faculty, other medical students not currently on the pediatric core clerkship). Data was collected daily approximately 24 hours after posting the Story from the previous day.

To consistently and systematically evaluate the data gathered, primary measures were defined as follows:

**Retention rate** = (number of Instagram users who viewed all slides in a Story ÷ number of

Instagram users who viewed the first slide in a Story) × 100

**Quiz engagement rate** = (number of Instagram users who answered the quiz question ÷

number of Instagram users who viewed the quiz question) × 100

**Percent correct on quiz** = (number of Instagram users who correctly answered the quiz

question ÷ number of Instagram users who answered the quiz question) × 100

At the end of each clerkship block, a voluntary and anonymous 7-item survey was distributed by institutional e-mail to all medical students on the current pediatric clerkship block using the Stony Brook University Qualtrics platform to assess their experiences with the Instagram Stories and account. The survey was developed by study investigators and included five questions using a 7-point scale where 1 = strongly disagree and 7 = strongly agree, as well as two open-ended questions for additional feedback. A positive rating was defined as responding strongly agree, agree, or somewhat agree to a survey item. Survey items were developed to inform direct feedback about the curriculum and specifically asked about feasibility, relevance to the clerkship, whether it increased clerkship engagement, if it helped prepare students for the NBME pediatric subject exam, and whether students would recommend this educational modality to other core clinical clerkships (Appendix). Qualitative analysis of two open-ended questions was conducted by study investigators (RL, CS, MC).

Coding of phrases and texts were performed inductively for identification of key themes. Students did not receive any compensation for participation and were able to participate in the curriculum regardless of their decision to complete the satisfaction survey. Demographic data was not collected to protect student anonymity and encourage honest responses to the survey questions without fear of academic consequences.

NBME pediatric subject exam performances for a one-year period prior to the Instagram Stories intervention as well as during the yearlong study period were measured. NBME total scores (expressed as percentage correct out of 100 scored questions) and corresponding percentiles were analyzed. Percentiles are provided by the NBME and updated on an annual basis based on aggregated national scores. A two-tailed unpaired t-test was conducted with significance level set at α = 0.05. Descriptive statistics and analysis were conducted using Microsoft Excel.

Curriculum design and reporting for this study followed the Guideline for Reporting Evidence-Based practice Educational Interventions and Teaching (GREET) checklist for reporting educational interventions [20]. This study was reviewed by our institutional Office of Research Compliance/CORIHS and determined to be a systematic investigation. As such, the study did not meet the definition of human subjects research according to the Common Rule (45 CFR 46 subpart A) and did not require approval by or exemption from the IRB. As instructed, the principal investigator (MC) obtained local departmental endorsement from the department chair as an authorized designee and ensured that the study complied with applicable regulations (i.e., FERPA) prior to beginning the investigation.

## Results

### Curricular Use and Learner Engagement

**Figure 2.**
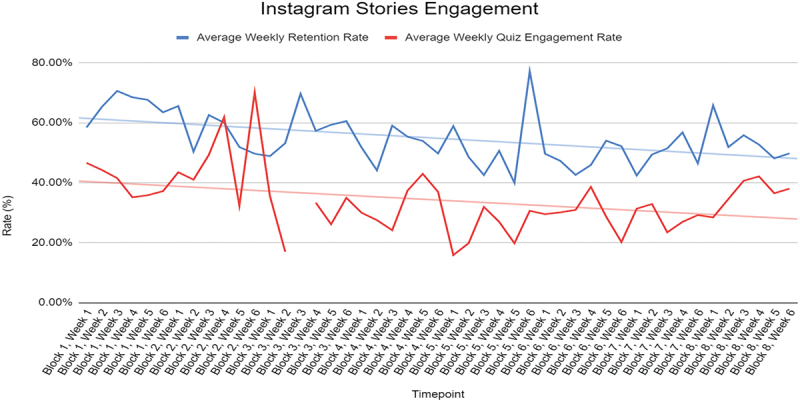
Pediatric Instagram Stories average weekly retention rate and quiz engagement rate over time. A full year of 6-week clerkship blocks is represented.

The study period spanned from May 2022 to July 2023. A total of 132 third-year medical students participated in the pediatric clerkship during this period and the average number of students in an individual clerkship block during the study period was 16.5. The average number of views on the first slide in a Story was 24.8, and the average number of views on the last slide of a Story was 13.3 per block. The average retention rate was 54.3%, the quiz engagement rate was 32.0%, and the percent correct on quiz was 87.6%. A graph of the average weekly retention rate and quiz engagement rate is shown in Figure 2. Sporadic data points were missed as they were not recorded during the 24-hour period Instagram allows Stories to be viewable and could not be retrieved.

### Learner Satisfaction

**Figure 3.**
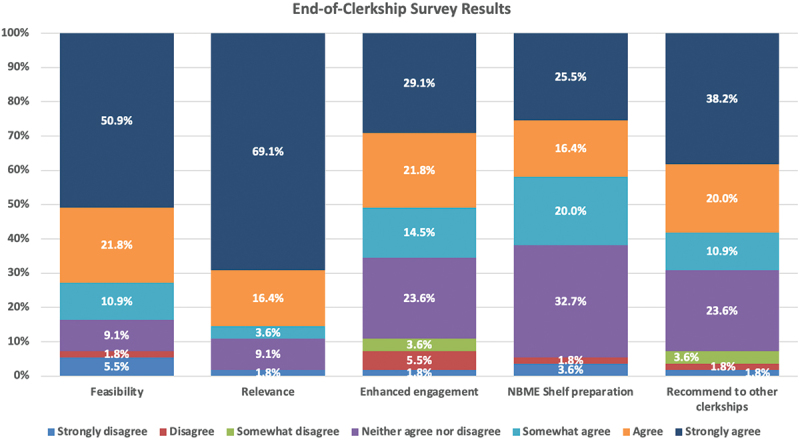
End-of-clerkship survey results (n = 55). Participants used a 7-point scale to rate the Instagram Stories project on feasibility, relevance to the clerkship, whether it enhanced clerkship engagement, its helpfulness in preparing for the NBME shelf exam, and whether it should be used in other clinical clerkships.

The voluntary satisfaction survey administered at the end of each clerkship block received a cumulative 55 responses with an overall response rate of 41.7%. Survey results are shown in Figure 3. Students positively rated the Instagram Stories curriculum on feasibility (83.6%), clerkship relevance (89.1%), facilitating engagement in the material (65.5%), assisting in NBME Shelf Exam preparation (61.8%), and recommending the modality to other clinical clerkships (69.1%).

**Figure 4.**
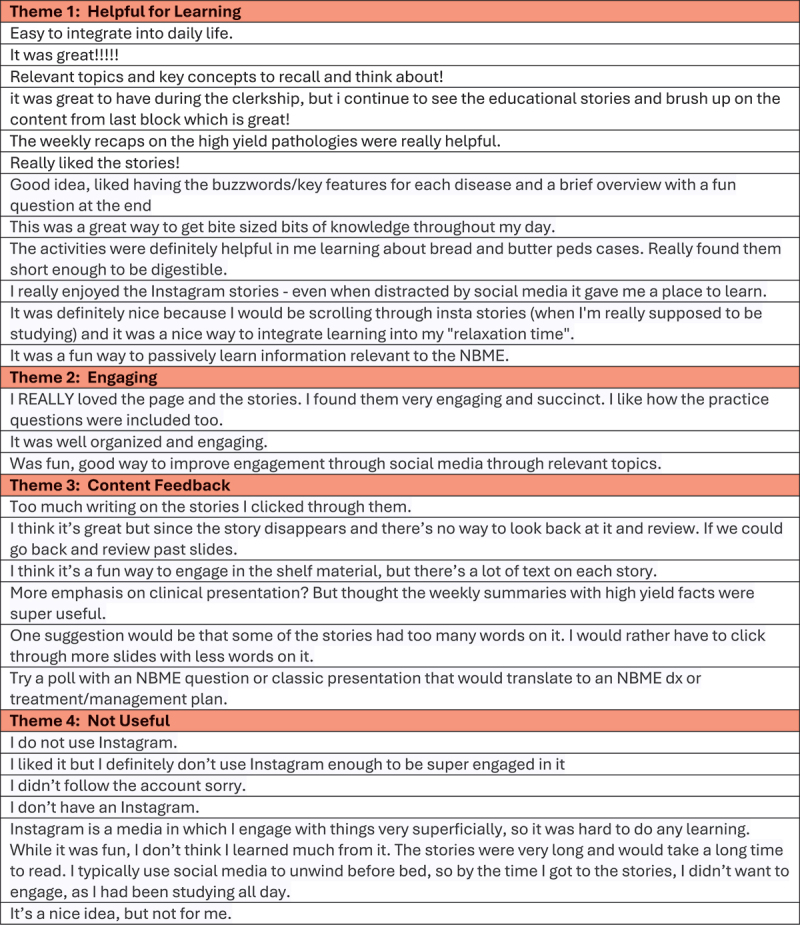
Themes from open-ended survey questions with example comments.

Qualitative analysis of open-ended responses regarding participants’ overall impression of the curriculum as well as recommendations for future directions provided constructive feedback for future iterations of the curriculum. Themes identified by investigators included content relevance/helpfulness for exam preparation, engagement, content feedback, and lack of use (Figure 4). Examples of specific positive comments from learners included ‘easy to integrate into daily life’, ‘makes me feel less guilty when procrastinating on social media’, ‘weekly summaries were very helpful’, and ‘great resource for bite-sized information’. Negative comments included ‘I do not have Instagram’, ‘I keep forgetting it exists so it’s hard to keep checking’, ‘slides were too wordy’, and ‘since the Story disappears there’s no way to look back and review’. The responses highlighted an interest in more complex high-yield topics that more closely mirror challenging NBME questions. Participants also asked for more quiz questions to assist with practice, mastery of the material, and NBME preparation. 16.7% of the open-ended responses indicated that the study participant did not have Instagram accounts or preferred to minimize and/or limit its use to social engagement.

There were no significant pre- and post-curricular implementation differences in the average NBME shelf exam scores (80.8 vs 81.2, *p-value* = 0.68) or corresponding percentile (59.9 vs 61.6, *p-value* = 0.62).

## Discussion

Integration of social media into medical education has shown promising results [7–18,21–31]. Curricular use of social media has been shown to bridge the gap and aid communication between educators and students [28,29]. Instagram, in particular, is extremely popular with medical students in the United States, both socially and professionally [5,7,18,21–24,28–31]. Successful creation of educational Instagram accounts has been described across a variety of clinical disciplines, including, but not limited to, radiology [9,10,15,17], obstetrics/gynecology [11], orthopedic surgery [13], and urology [16]. Our study provides evidence that the use of the Instagram Story modality as an educational adjuvant in a core clinical clerkship is feasible, engaging, relevant, and appreciated by medical students. Satisfaction survey items regarding feasibility and relevance to the pediatrics rotation were the most favorably rated by study participants. The positive ratings in the feasibility category suggested that the curriculum was accessible for a majority of participants and did not require a large expenditure of time. This was confirmed with the open-ended responses for feedback. 16.6% of responses to the feasibility item in the ‘neither agree not disagree’, ‘somewhat disagree’, ‘disagree’ or ‘strongly disagree’ categories approximated the percentage of responses indicating neutrality or lack of curriculum engagement from the open-ended survey questions. We did not have any way to correlate those neutral or negative responses with curriculum engagement as we did not directly ask about personal use of Instagram on the survey. Therefore, it is difficult to determine if those responses were due to an unfavorable opinion of the curriculum itself or reflected a lack of access to the curriculum due to its access only through the Instagram platform.

The positive response to relevance to the rotation survey item (89.1%) provided investigators with encouraging feedback about the content selected for inclusion. While a majority of students felt that the curriculum helped with NBME examination preparation (61.8%), more reported ‘neutral’ responses than with the other questions, leaving us to conclude that the curriculum may have been considered more helpful from a clinical perspective rather than for exam preparation. The Stories were commended for the high yield topics and bite-sized educational content in open-ended response survey questions. Content was criticized for excessive text on the graphics and inability to re-visit the Story after it expired. Modifications were made to address this feedback as the curriculum progressed. Although Instagram Stories expire after 24 hours, we were able to save content in the account as a Story highlight so users would be able to access a library of content as needed. Review of existing literature suggests that students prefer the educational use of social media for quizzes and clarification/explanation of curricular material [25,26,32]. Participant responses in our study supported this conclusion as well, with the mini-quiz questions at completion of each Story and summary quizzes at the end of each week receiving positive reception in particular.

To date, the majority of studies published have been cross-sectional/observational with primary aims of measuring usage and engagement to determine how social media use can benefit educational practices rather than outcomes [13]. Platform engagement has been assessed through measurements of followers, views, and likes [7–11], whereas we endeavored to further define engagement by measuring user retention throughout a particular story as well as quiz completion rates. It is interesting to note that each Story typically received more views than the number of students on clerkship in any individual block, suggesting the possibility that some learners remained engaged in the curriculum even after completion of the clerkship. This presented an unintended limitation to analysis of usage and engagement per block in that faculty members, study team members, other medical students not currently enrolled in the pediatric clinical clerkship, and even members of the general public were often noted to be viewing the Stories and interacting with quiz questions. This phenomenon has been reported in other fields as well; a study of Instagram use in obstetrics/gynecology at the University of Michigan created a public Instagram account for educational purposes [10] and found that 77.8% of account users self-reported as medical students, with email addresses from thirty different US institutions, exhibiting the potential wide reach of a public educational Instagram account.

Beyond engagement and learner satisfaction measures, investigators have struggled to assess the impact of social media use in medical education regarding knowledge acquisition, skills, or behaviors [7–24,32]. A 2013 systematic review of literature regarding the use of social networking sites in medical education did not find any solid evidence that the use of social media is equivocal or superior to other resources for learning [17]. Saade et al. surveyed urologists regarding their perceptions of knowledge acquisition and found that social media was the third most popularly identified resource after academic journals and society websites, with a majority of respondents rating its perceived influence as moderate or high [15]. Investigators conclude that there is a paucity of outcome-based, empirical studies to specifically address the educational impact on undergraduate medical education [18].

Acknowledging that NBME shelf exam preparation is individualized and complex, we recognized that this curriculum would not be utilized as a primary preparatory material for students. The students in the pediatric clerkship at our institution generally perform well on the shelf exam, with an average of 60% of students scoring above the 50th percentile based on national standards; therefore, we were not expecting to see a meaningful direct impact on shelf exam performance. The small increase in the average shelf exam score between pre- and post-curricular groups was an interesting finding, despite the lack of statistical significance. It is conceivable that the Instagram content served as a reminder or impetus for clerkship students to engage in other preparatory behaviors. We conclude that further investigation should be undertaken to determine the potential impact of an Instagram curriculum on summative examination performance.

## Limitations

We identified several limitations with this study. Although the study team made every effort to consistently post Stories at the same time each day (approximately 8:00 A.M. local time), there were many days where the study team was unable to post content until later in the day due to clinical obligations. Maintenance of the account required a daily commitment from study investigators which proved challenging at times. While not deliberately measured, investigators observed subjective declines in Story engagement on days when Stories were posted at later times, perhaps indicating that users in our clerkship did not access their social media accounts later in the day as widely as in the morning. More extensive review of user social media practices would further inform this finding. Future research and efforts in this type of educational intervention would benefit from consistent posting and data collection strategies to remove confounding variables.

Since the satisfaction survey was developed by study investigators to answer specific questions about the curriculum, we neglected to include a question asking study participants if they utilized Instagram. We were surprised to find that 16.7% of the open-ended responses indicated learners did not use or preferred not to use Instagram for professional reasons. This represented a significant number of learners who could not or would not access this resource, thereby limiting the overall effectiveness of the intervention.

## Conclusions

The evolution of social media within Web 2.0 offers educators the opportunity to engage learners in a contemporary form of self-directed learning. Use of social media within medical education can stimulate reflection, foster professional identify formation, and encourage students to be active in knowledge acquisition and synthesis [27,33]. Based on the results of our study, we conclude that the social media platform Instagram can be successfully integrated into medical education endeavors and should be strongly considered as a supplemental tool to aid learners and modernize clinical curricula. Survey responses demonstrated widespread approval of the curriculum from students specific to clerkship relevance and feasibility. Considerate integration of Instagram Stories in core clinical clerkships can have positive impact on outcomes beyond summative examinations, including the learning environment, student curiosity, intrinsic motivation, and problem-solving skills [33].

## Future steps

Educational interventions using social media platforms like Instagram should be pursued and outcomes evaluated to determine if meaningful impact on knowledge acquisition, skills, or behaviors can be demonstrated. Further investigation and summary of best practices for Instagram Story use in medical education both in design and in implementation would be helpful for future content creators [29]. To date, surveys of medical students regarding educational posts found that those containing quizzes, explanatory comments, post-lecture questions, book/article recommendations, and instructional videos were most preferred [29]. Student perception effectiveness of social media use in medical education is linked to ease of accessibility and length of content, shorter is better [32]. Our study findings aligned with this as well. Ultimately, when considering use of social media-based educational interventions, educators should engage their learners in the design process to maximize successful implementation.

## Data Availability

The datasets used and/or analyzed during the current study are available from the corresponding author on reasonable request.

## Abbreviations

UME: Undergraduate Medical Education
NBME: National Board of Medical Examiners

## Authors’ information

Drs. Bellis, Kim and Sniffen were senior medical students during their participation enrollment and curriculum implementation for this study as part of a longitudinal teaching and leadership elective at Renaissance School of Medicine at Stony Brook University. Drs. Lendway and Smith are pediatric residents at Stony Brook Children’s. Dr. Chitkara is a Professor of Pediatrics, Chief of Pediatric Hospital Medicine, and Director of Undergraduate Medical Education in Pediatrics at Renaissance School of Medicine at Stony Brook University.
